# Supplementary material for: DunedinPACE, a DNA methylation biomarker of the pace of aging
Source: eLife. 2022 Jan 14;11:e73420. doi: 10.7554/eLife.73420 (PMC8853656; doi:10.7554/eLife.73420)
Supplement: Supplementary file 1. — (A) Measurements included in modeling Pace of Aging in the Dunedin Study Birth Cohort. We measured Pace of Aging from repeated assessments of a panel of 19 biomarkers: Body mass index (BMI), Waist-hip ratio, Glycated hemoglobin, Leptin, Blood pressure (mean arterial pressure), Cardiorespiratory fitness (VO2Max), Forced vital capacity ratio (FEV1/FVC), Forced expiratory volume in one second (FEV1), Total cholesterol, Triglycerides, High density lipoprotein (HDL), Lipoprotein(a), Apolipoprotein B100/A1 ratio, estimated Glomerular Filtration Rate (eGFR), Blood Urea Nitrogen (BUN), High Sensitivity C-reactive Protein (hs-CRP), White blood cell count, mean periodontal attachment loss (AL), and the number of dental-caries-affected tooth surfaces (tooth decay). This list includes two new biomarkers, leptin and caries, not included in the original Pace of Aging (Belsky et al., 2015), both of which have now been assessed at multiple waves, allowing growth curve modeling. Telomere length was dropped because of an emerging and yet-unresolved field-wide debate about its measurement (Nettle et al., 2021). All other biomarkers were the same. The 20 year Pace of Aging measure is described in detail elsewhere (Elliott et al., 2021b). (B) CpGs included in the DunedinPACE DNA methylation algorithm, their mean values in the Dunedin Study, and the coefficients for their weights in the DunedinPACE algorithm. (C) Independent associations of DunedinPACE with mortality, morbidity, and disability in models including covariate adjustment for DNA methylation clocks. The table reports effect-sizes for DunedinPACE and DNA methylation clocks from time-to-event analysis of mortality, cardiovascular disease (CVD), and stroke or transient ischemic attack (TIA) and repeated-measures analysis of incident limitations to activities of daily living (ADLs) in the Framingham Heart Study Offspring cohort. Each model includes DunedinPACE, one of the DNA methylation clocks, and covariates for age and sex. Ti [file elife-73420-supp1.docx]

**Supplementary File 1A. Measurements included in modeling Pace of Aging in the Dunedin Study Birth Cohort**. We measured Pace of Aging from repeated assessments of a panel of 19 biomarkers: Body mass index (BMI), Waist-hip ratio, Glycated hemoglobin, Leptin, Blood pressure (mean arterial pressure), Cardiorespiratory fitness (VO_2_Max), Forced vital capacity ratio (FEV_1_/FVC), Forced expiratory volume in one second (FEV_1_), Total cholesterol, Triglycerides, High density lipoprotein (HDL), Lipoprotein(a), Apolipoprotein B100/A1 ratio, estimated Glomerular Filtration Rate (eGFR), Blood Urea Nitrogen (BUN), High Sensitivity C-reactive Protein (hs-CRP), White blood cell count, mean periodontal attachment loss (AL), and the number of dental-caries-affected tooth surfaces (tooth decay). This list includes two new biomarkers, leptin and caries, not included in the original Pace of Aging (Belsky et al., 2015), both of which have now been assessed at multiple waves, allowing growth curve modeling. Telomere length was dropped because of an emerging and yet-unresolved field-wide debate about its measurement (Nettle et al., 2021). All other biomarkers were the same. The 20-year Pace of Aging measure is described in detail elsewhere (Elliott et al., 2021).

| ***Body mass index*** | Height was measured to the nearest millimeter using a Seca 264 Wireless Stadiometer. Weight was measured to the nearest 0.1 kg using calibrated scales. Individuals were weighed in light clothing. Body mass index (BMI) was calculated using the standard formula weight in kilograms divided by height in meters squared. |
| --- | --- |
| ***Waist-hip ratio*** | Waist girth was the perimeter at the level of the noticeable waist narrowing located between the costal border and the iliac crest. Hip girth was taken as the perimeter at the level of the greatest protuberance and at about the symphysion pubic level anteriorly. Measurements were repeated and the average used to calculate waist-hip ratio. |
| ***Glycated hemoglobin (HbA1C)*** | Whole blood glycated hemoglobin concentration (expressed as a percentage of total hemoglobin) was measured by ion exchange high performance liquid chromatography (BioRad D-100, Hercules, Calif.), a method certified by the US National Glycohemoglobin Standardization Program (<http://www.ngsp.org/>). |
| ***Leptin*** | Serum leptin (μg/L) was measured using Human Leptin RIA kit (Cat# HL-81K, Linco Research, Missouri, USA) (Ages 32 & 38) and the Quantikine ELISA Human Leptin Immunoassay (Cat# SLP00, R&D Systems Inc, Minneapolis, MN) (Age 45) according to the manufacturer’s instructions. |
| ***Blood pressure (mean arterial pressure)*** | Systolic and diastolic blood pressure were assessed according to standard protocols with a Hawksley random-zero sphygmomanometer with a constant deflation valve (age 32 & 38) and with a BpTRU™ Vital Signs Monitor BPM 200 (age 45). Mean arterial pressure (MAP) was calculated using the formula Diastolic Pressure+1/3(Systolic Pressure - Diastolic Pressure). |
| ***Cardiorespiratory fitness (VO_2_Max)*** | Cardiorespiratory fitness was assessed by measuring heart rate in response to a submaximal exercise test on a friction-braked cycle ergometer. Dependent on the extent to which heart rate increased during a 2-min 50 W warm-up, the workload was adjusted to elicit a steady heart-rate in the range 130–170 beats per minute. After a further 6-min constant power output stage, the maximum heart rate was recorded and used to calculate predicted maximum oxygen uptake adjusted for body weight in milliliters per minute per kilogram (VO_2_max) according to standard protocols. |
| ***Lung function (FEV_1_ and*** ***FEV_1_/FVC)*** | We calculated post-albuterol forced expiratory volume in one second (FEV_1_) and the ratio of FEV_1_ to forced vital capacity (FVC; FEV_1_/FVC) using measurements from spirometry conducted with a Sensormedics body plethysmograph (Sensormedics Corporation, Yorba Linda, CA, USA). |
| ***Total cholesterol, triglycerides, and high-density lipoprotein (HDL) cholesterol*** | Serum non-fasting total cholesterol, triglycerides, and high-density lipoprotein (HDL) cholesterol levels (mmol/L) were measured by colorimetric assay on a Hitachi 917 analyzer (ages 26-32), a Modular P analyzer (age 38), and a Cobas c702 analyzer (age 45). |
| ***Lipoprotein(a)*** | Serum lipoprotein(a) (nmol/L) was measured by a particle-enhanced immunoturbidimetric assay on a Hitachi 917 analyzer (ages 26-32), a Modular P analyzer (age 38), and a Cobas c502 analyzer (age 45). |
| ***Apolipoprotein B100/A1 ratio*** | Serum apolipoprotein A1 (g/L) and apolipoprotein B100 (g/L) were measured by immunoturbidimetric assay on a Hitachi 917 analyzer (ages 26-32), a Modular P analyzer (age 38), and a Cobas c502 (age 45), and the ratio between the two was calculated. |
| ***eGFR (estimated glomerular filtration rate)*** | Serum creatinine (umol/L) was measured by kinetic colorimetric assay on a Hitachi 917 analyzer (age 32), Modular P analyzer (age 38), and Cobas c702 (age 45) (Roche Diagnostics, Mannheim, Germany). eGFR was estimated utilizing the CKD-Epi formula calculated from serum creatinine. |
| ***Blood urea nitrogen (BUN)*** | Blood urea nitrogen (mmol/L) was measured by kinetic UV assay at ages 26 (Hitachi 917 analyzer) and 45 (Cobas c702 analyzer), and by kinetic colorimetric assay at ages 32 (Hitachi 917 analyzer) and 38 (Modular P analyzer), , and Cobas c702 analyzer at age 45. |
| ***High sensitivity C-reactive protein (hs-CRP)*** | Serum C-reactive protein (mg/L) was measured by high sensitivity immunoturbidimetric assay on a Hitachi 917 analyzer (age 32), a Modular P analyzer (age 38), and a Cobas c702 (age 45). Values were log-transformed for analysis. |
| ***White blood cell count*** | Whole blood white blood cell counts (x10^9^/L) were measured by flow cytometry with a Coulter STKS (Coulter Corporation, Miami, FL) (age 26), a Sysmex XE2100 (Sysmex Corporation, Japan) (age 32), and a Sysmex XE5000 (Sysmex Corporation, Japan) (ages 38 and 45). Counts were log-transformed for analysis. |
| ***Mean periodontal attachment loss (AL)*** | Calibrated dentists used a PCP-2 periodontal probe (Hu-Friedy; Chicago) to measure gingival recession (the distance from the cementoenamel junction to the gingival margin) and probing depth (the distance from the probe tip to the gingival margin) in millimeters at three sites (mesiobuccal, buccal, and distolingual) per tooth, excluding third molar teeth. Periodontal attachment loss for each site was computed by summing gingival recession and probing depth. and then averaged across all periodontally examined teeth. Periodontal examinations were conducted with half-mouth examinations at age 26 and full-mouth examinations at ages 32, 38, and 45 years. |
| ***Caries-affected tooth surfaces*** | Calibrated dentists examined the teeth for caries and restorations following the World Health Organization Oral Health Surveys methodology. Four surfaces were considered for anterior teeth (canines and incisors): buccal, lingual, distal, and mesial; a fifth surface, occlusal, was considered for premolar and molar teeth. Tooth surfaces were classified as having untreated caries (DS) if a cavitated carious lesion was present, as filled (FS) if a dental restoration was present (including crowns), and missing due to caries (MS) if the participant indicated that a given tooth had been removed due to decay or failed dental restorative work. DS, MS, and FS counts were summed to obtain a DMFS score (ranging from 0 to 148 surfaces). Caries experience was expressed as the % of tooth surfaces that had been caries-affected, excluding surfaces of teeth that were unerupted, lost due to trauma, extracted for reasons other than caries (impaction, orthodontic treatment, or periodontal disease), or could not be visualized by the examiner. |

**Supplementary File 1B. CpGs included in the DunedinPACE algorithm and their scoring coefficients.** CpG IDs and scoring coefficients are provided for documentation only. Computation of the DunedinPACE measure from array data requires additional data normalization performed within the R package DunedinPACE, available from GitHub at <https://github.com/danbelsky/DunedinPACE>.

**Supplementary File 1C. Independent associations of DunedinPACE with mortality, morbidity, and disability in models including covariate adjustment for DNA methylation clocks.** The table reports effect-sizes for DunedinPACE and DNA methylation clocks from time-to-event analysis of mortality, cardiovascular disease (CVD), and stroke or transient ischemic attack (TIA) and repeated-measures analysis of incident limitations to activities of daily living (ADLs) in the Framingham Heart Study Offspring cohort. Each model includes DunedinPACE, one of the DNA methylation clocks, and covariates for age and sex. Time-to-event model effect-sizes are reported as hazard ratios (HR). Repeated-measures model effect-sizes are reported as incidence-rate ratios (IRR).

**Supplementary File 1D. Physical and cognitive functioning and subjective signs of aging measures in the Dunedin Study**

| **Physical Functioning** (N=817 with DNAm data) | |
| --- | --- |
| Balance | Balance was measured using the Unipedal Stance Test as the maximum time achieved across three trials of the test with eyes closed (Bohannon et al., 1984; Springer et al., 2007; Vereeck et al., 2008). |
| Gait Speed | Gait speed (meters per second) was assessed with the 6-m-long GAITRite Electronic Walkway (CIR Systems, Inc) with 2-m acceleration and 2-m deceleration before and after the walkway, respectively. Gait speed was assessed under 3 walking conditions: usual gait speed (walk at normal pace from a standing start, measured as a mean of 2 walks) and 2 challenge paradigms, dual-task gait speed (walk at normal pace while reciting alternate letters of the alphabet out loud, starting with the letter “A,” measured as a mean of 2 walks) and maximum gait speed (walk as fast as safely possible, measured as a mean of 3 walks). We calculated the mean of the 3 individual walk conditions to generate our primary measure of composite gait speed (Rasmussen et al., 2019). |
| Steps in Place | The 2-min step test was measured as the number of times a participant lifted their right knee to mid-thigh height (measured as the height half-way between the knee cap and the iliac crest) in 2 minutes at a self-directed pace (Jones and Rikli, 2002; Rikli and Jones, 1999). |
| Chair Stands | Chair rises were measured as the number of stands a participant completed in 30 seconds from a seated position (Jones et al., 1999; Jones and Rikli, 2002). |
| Grip Strength | Handgrip strength was measured for the dominant hand (elbow held at 90°, upper arm held tight against the trunk) as the maximum value achieved across three trials using a Jamar digital dynamometer (Mathiowetz et al., 1985; Rantanen T et al., 1999). |
| Motor Coordination | At ages 38 and 45, we measured motor functioning as the time to completion of the Grooved Pegboard Test with the dominant hand. |
| Physical Limitations | Physical limitations were measured with the 10-item RAND 36-Item Health Survey 1.0 physical functioning scale (Ware and Sherbourne, 1992). Participant responses (“limited a lot”, “limited a little”, “not limited at all”) assessed their difficulty with completing various activities, e.g., climbing several flights of stairs, walking more than 1 km, participating in strenuous sports, etc. Scores were reversed to reflect physical limitations so that a high score indicates more limitations. |
| Decline in Physical Functioning | Tests of balance and grip strength and interviews about physical limitations were completed at both the age-38 and age-45 Dunedin Study assessments. We measured decline across the 7-year measurement interval by subtracting the age-38 test score from the age-45 test score. |
| **Cognitive Functioning** (N=814 with DNAm data) | |
| Cognitive Functioning | The Wechsler Adult Intelligence Scale-IV (WAIS-IV) (Wechsler, 2008) was administered to the participants at age 45 years, yielding the IQ. In addition to full scale IQ, the WAIS-IV measures four specific domains of cognitive function: Processing Speed, Working Memory, Perceptual Reasoning, and Verbal Comprehension. |
| Cognitive Decline | IQ is a highly reliable measure of general intellectual functioning that captures overall ability across differentiable cognitive functions. We measured IQ from the individually administered Wechsler Intelligence Scale for Children-Revised (WISC-R; averaged across ages 7, 9, 11, and 13) (Wechsler, 2003) and the Wechsler Adult Intelligence Scale-IV (WAIS-IV; age 45) (Wechsler, 2008). We measured IQ decline by comparing scores from the WISC-R and the WAIS-IV. |
| **Subjective Signs of Aging** (N=817 with DNAm data) | |
| Self-rated Health | Study members rated their health on a scale of 1-5 (poor, fair, good, very good, or excellent). |
| Facial Aging | Facial Aging is the subjective perception of aged appearance based on a facial photograph and is proposed as a clinically-useful marker of mortality risk (Christensen et al., 2009). Facial Aging measurement in the Dunedin Study was based on ratings by an independent panel of 8 raters of each participant’s facial photograph (Belsky et al., 2015; Shalev et al., 2014). Facial Aging was based on two measurements of perceived age. First, Age Range was assessed by an independent panel of 4 raters, who were presented with standardized (non-smiling) facial photographs of participants and were kept blind to their actual age. Raters used a Likert scale to categorize each participant into a 5-year age range (i.e., from 20-24 years old up to 70+ years old) (interrater reliability = .77). Scores for each participant were averaged across all raters. Second, Relative Age was assessed by a different panel of 4 raters, who were told that all photos were of people aged 45 years old. Raters then used a 7-item Likert scale to assign a “relative age” to each participant (1=“young looking”, 7=“old looking”) (interrater reliability = .79). The measure of perceived age at 45 years, Facial Age, was derived by standardizing and averaging Age Range and Relative Age scores. |
| Subjective Decline | Self-rated Health and Facial Aging were measured at both the age-38 and age-45 assessments. We measured decline in self-rated health as incident fair/poor health reported at the age-45 assessment. We measured acceleration in Facial Aging by computing the difference in Facial Aging Z-scores between the age-45 and age-38 assessments. |

**Supplementary File 1E. Items included in Nagi, Katz, and Rosow-Breslau scales of limitations to Activities of Daily Living (ADLs).**

| **Nagi ADL Scale.** Count of activities for which participants reported a lot of difficulty or inability to perform. |
| --- |
| Pulling or pushing large objects |
| Stooping, crouching, or kneeling |
| Reaching or extending arms below shoulder level |
| Reaching or extending arms above shoulder level |
| Writing, handling, or fingering small objects |
| Standing in one place for long periods (15 minutes) |
| Sitting for long periods (one hour) |
| Lifting or carrying weights under 10 lbs |
| Lifting or carrying weights over 10 lbs |
| **Katz ADL Scale.** Count of activities for which participants required assistance or could not do themselves. |
| Dressing |
| Bathing |
| Eating |
| Transferring (getting in and out of a chair) |
| Toileting |
| **Rosow-Breslau ADL Scale.** Count of activities participants were not able to do. |
| Heavy work around the house |
| Walk half a mile without assistance |
| Walk up and down one flight of stairs |

**SUPPLEMENT REFERENCES**

Belsky DW, Caspi A, Houts R, Cohen HJ, Corcoran DL, Danese A, Harrington H, Israel S, Levine ME, Schaefer JD, Sugden K, Williams B, Yashin AI, Poulton R, Moffitt TE. 2015. Quantification of biological aging in young adults. *Proc Natl Acad Sci USA* **112**:E4104-4110. doi:10.1073/pnas.1506264112

Bohannon RW, Larkin PA, Cook AC, Gear J, Singer J. 1984. Decrease in timed balance test scores with aging. *Phys Ther* **64**:1067–1070.

Christensen K, Thinggaard M, McGue M, Rexbye H, Hjelmborg J v B, Aviv A, Gunn D, van der Ouderaa F, Vaupel JW. 2009. Perceived age as clinically useful biomarker of ageing: cohort study. *BMJ* **339**. doi:10.1136/bmj.b5262

Elliott ML, Caspi A, Houts RM, Ambler A, Broadbent JM, Hancox RJ, Harrington H, Hogan S, Keenan R, Knodt A, Leung JH, Melzer TR, Purdy SC, Ramrakha S, Richmond-Rakerd LS, Righarts A, Sugden K, Thomson WM, Thorne PR, Williams BS, Wilson G, Hariri AR, Poulton R, Moffitt TE. 2021. Disparities in the pace of biological aging among midlife adults of the same chronological age have implications for future frailty risk and policy. *Nat Aging* **1**:295–308. doi:10.1038/s43587-021-00044-4

Jones CJ, Rikli RE. 2002. Measuring functional fitness in older adults. *The Journal on active aging* **1**:24–30.

Jones CJ, Rikli RE, Beam WC. 1999. A 30-s chair-stand test as a measure of lower body strength in community-residing older adults. *Research quarterly for exercise and sport* **70**:113–119.

Mathiowetz V, Kashman N, Volland G, Weber K, Dowe M, Rogers S. 1985. Grip and pinch strength: normative data for adults. *Arch Phys Med Rehabil* **66**:69–74.

Nettle D, Gadalla SM, Lai T-P, Susser E, Bateson M, Aviv A. 2021. Telomere length measurement for longitudinal analysis: implications of assay precision. *Am J Epidemiol*. doi:10.1093/aje/kwab025

Rantanen T, Guralnik JM, Foley D, et al. 1999. MIdlife hand grip strength as a predictor of old age disability. *JAMA* **281**:558–560. doi:10.1001/jama.281.6.558

Rasmussen LJH, Caspi A, Ambler A, Broadbent JM, Cohen HJ, d’Arbeloff T, Elliott M, Hancox RJ, Harrington H, Hogan S, Houts R, Ireland D, Knodt AR, Meredith-Jones K, Morey MC, Morrison L, Poulton R, Ramrakha S, Richmond-Rakerd L, Sison ML, Sneddon K, Thomson WM, Hariri AR, Moffitt TE. 2019. Association of Neurocognitive and Physical Function With Gait Speed in Midlife. *JAMA Netw Open* **2**:e1913123–e1913123. doi:10.1001/jamanetworkopen.2019.13123

Rikli RE, Jones CJ. 1999. Functional fitness normative scores for community-residing older adults, ages 60-94. *Journal of aging and physical activity* **7**:162–181.

Shalev I, Caspi A, Ambler A, Belsky DW, Chapple S, Cohen HJ, Israel S, Poulton R, Ramrakha S, Rivera CD, Sugden K, Williams B, Wolke D, Moffitt TE. 2014. Perinatal complications and aging indicators by midlife. *Pediatrics* **134**:e1315-1323. doi:10.1542/peds.2014-1669

Springer BA, Marin R, Cyhan T, Roberts H, Gill NW. 2007. Normative values for the unipedal stance test with eyes open and closed. *J Geriatr Phys Ther* **30**:8–15.

Thomas S. 2017. Telomeres as Sentinels for Environmental Exposures, Psychosocial Stress, and Disease Susceptibility: Workshop Summary. *NIEHS-NIA Workshop on Telomeres*.

Vereeck L, Wuyts F, Truijen S, Van de Heyning P. 2008. Clinical assessment of balance: Normative data, and gender and age effects. *International Journal of Audiology* **47**:67–75. doi:10.1080/14992020701689688

Ware JE Jr, Sherbourne CD. 1992. The MOS 36-Item Short-Form Health Survey (SF-36): I. Conceptual Framework and Item Selection. *Medical Care* **30**:473–483.

Wechsler D. 2008. Wechsler Adult Intelligence Scale, 4th ed. San Antonio, TX: Pearson Assessment.

Wechsler D. 2003. Wechsler Intelligence Scale for Children, 4th (UK Version). ed. San Antonio, TX: Harcourt Assessment.
